# Suitability of a new Bloom filter for numerical vectors with high dimensions

**DOI:** 10.1371/journal.pone.0209159

**Published:** 2018-12-21

**Authors:** Chunyan Shuai, Jiayou Lei, Zeweiyi Gong, Xin Ouyang

**Affiliations:** 1 Faculty of Transportation Engineering, Kunming University of Science and Technology, Kunming, China; 2 Faculty of Electric Power Engineering, Kunming University of Science and Technology, Kunming, China; 3 Faculty of Information Engineering and Automation, Kunming University of Science and Technology, Kunming, China; University of Electronic Science and Technology of China, CHINA

## Abstract

The notable increase in the size and dimensions of data have presented challenges for data storage and retrieval. The Bloom filter and its generations, due to efficient space overheads and constant query delays, have been broadly applied to querying memberships of a big data set. However, the Bloom filter and most of the variants regard each element as a 1-dimensional string and adopt multiple different string hashes to project the data. The interesting problem is when the inputs are numerical vectors with high dimensions, it remains unknown whether they can be projected into the Bloom filter in their original format. Furthermore, we investigate whether the projection is random and uniform. To address these problems, this paper presents a new uniform Prime-HD-BKDERhash family and a new Bloom filter (P-HDBF) to retrieve the membership of a big data set with the numerical high dimensions. Since the randomness and uniformity of data mapping determines the performance of the Bloom filter, to verify these properties, we first introduce information entropy. Our theoretical and experimental results show that the P-HDBF can randomly and uniformly map the data in their native formats. Moreover, the P-HDBF provides an efficient solution alternative to implement membership search with space-time overheads. This advantage may be suitable for engineering applications that are resource-constrained or identification of the nuances of the graphics and images.

## 1. Introduction

With increasing data sizes, concise data representations and efficient query algorithms have become the key factors to large-scale data management. As a result, a large number of technologies have appeared, such as the Bloom filter (BF) [1]. The BF has a low query delay and a high time-space overhead, leading to its broad use in computing areas, such as network and network security [2–5], distributed systems [6–9] and applications or embedded devices [10,11], with limited computing and storage resources. Moreover, many variants have been proposed, including the counting Bloom filter (CBF) [12] and its improvements [13–14], the compressed Bloom filter[15], the spectral Bloom filter[16], the dynamic Bloom filter [17], the Cuckoo Filter[18], and the parallel BFs (PBF-HT and PBF-BF) [19, 20].

The BF can perform well and obtain a low false positive probability (FPP) only when the hash randomly and uniformly disperses the data, and usually, string hash functions [21] are the default choices. Regardless of the data format, the string hash takes the input as a 1-dimensional string, rather than its original format, and iteratively computes every character to obtain a random integer. To better scatter the data into different places and reduce the FPP, multiple different string hashes are usually selected. To project numerical vectors with high dimensions in their original formats, LshBFs [22–25] replace the string hashes with a uniform locality sensitive hashing (LSH) [26]. However, since the LSH gathers the data around the mean, LshBFs are more suitable for approximate nearest neighbours queries, rather than membership queries.

When the inputs are numerical vectors with high dimensions, this paper proposes dealing with them in their original formats other than strings. First, a unified prime BKDERhash [27] function family, denoted as Prime-HD-BKDERhash, is proposed to substitute for multiple different string hashes. Meanwhile, information entropy is introduced in the BF to verify the randomness and uniformity of the data mapped by the Prime-HD-BKDERhash. Next, by combining the unified Prime-HD-BKDERhash with a counter array, a new BF called P-HDBF is established to store and retrieve the memberships of the big data set. The theoretical analysis and experiments show that the Prime-HD-BKDERhash can disperse elements more effectively than the string hashes, and the P-HDBF is more suitable to represent and query the numerical vectors of a big data set in high-dimensional spaces, which has low space-time costs. Compared with the PBF-HT and PBF-BF, the P-HDBF possesses low false detection rates, low query delays and low space requirements. The advantages of the constant query delay and low space-time costs make the P-HDBF more appropriate for some engineering applications with constrained computing and storage resources, such as distinguish the nuances of the graphics and images.

The remainder of this paper is organized as follows. Related works are described in section 2. The design of Bloom filter and our structure are presented in section 3. The theoretical analyses and proofs are in Sections 4 and 5. Section 6 presents the related performance evaluation and experiments. Section 7 presents the study’s conclusions.

## 2. Related work

This section provides a brief survey related to the Bloom filter designs and its variants that are suitable for element deletion and multi-dimensional vectors.

A Bloom filter [1] utilities a slightly array to store a big data set. This filter uses the mappings of multiple string hashes to answer whether a query is member of the set with a small false positive probability or not. To support element deletion, the counting Bloom filter (CBF) [12] proves that a 4-bit counter array will be sufficient to defend against overflows brought by element deletion. The FPP, array size and cardinality of the BF have been discussed in [28–30]. The variable incremental counting Bloom filter (VI-CBF) [31] increases the counter by a variable increment rather than the unaltered increment to reduce memory costs. Moreover, with the same counter width, the query in VI-CBF can get a more complete answer than in CBF. The Cuckoo filter [18] consists of an array of buckets where each item has two candidate buckets. The filter computes every item's two fingerprints and bucket positions using hash functions *h*_1_(*x*) = *h*(*x*) and *h*_2_(*x*) = *h*_1_(*x*)⊕*h* (*h* is x's fingerprint). The lookup procedure checks both buckets to see if either one contains the query to determine the membership. Since the insert procedure will continuously relocate existing fingerprints to their alternatives until no more buckets can be allocated, it efficiently reduces the memory costs but results in a long computational time.

Bloom-1 [32] achieves a reduced query overhead at the cost of a higher FPP for a given memory size. Reviriego [33] provides a correct analysis of Bloom-1 and gives out an exact FPP. For the fixed FPP and cardinality of a dataset, the spaces that a BF required are determined. Once a number of extra elements are added in, the FPP will increase quickly. Therefore, the traditional BF is suitable for static sets. The Spectral BF [16] and Dynamic BF (DBF) [17] extend the BF to multi-set and dynamic sets, respectively. To determine which BF an element belongs to in cloud environment, Bloofi [34] organizes different BFs in a hierarchical index structure similar to a B+ tree and the FPP of the hierarchical Bloofi is discussed in [35].

These BFs recognize the inputs as 1-dimensional strings. PBF [20], PBF-HT and PBF-BF [21] have been developed to store and query multi-dimensional elements. The PBF consists of multiple parallel standard BFs, and each standard BF represents an attribute. Due to the destruction of the integrity of the attributes, the PBF generates a high FPP. Furthermore, to reduce the FPP, the PBF-HT (PBF-BF) adds a hash table (a check BF) to the PBF. Let *d* be the number of dimensions, let *m*_1_ and *m*_2_ be the sizes of the array of the BF and the HT (or the checkBF), and let *k*_1_ and *k*_2_ be numbers of hash functions of the PBF and the HT (or the check BF), respectively. The memory cost and query delay of the PBF-BF (or PBF-HT) are *dm*_1_+*m*_2_ and *k*_1_*d*+*k*_2_, respectively. Both of them grow linearly as dimensions increase and result in huge memory wastes and query delays. Rather than applying multiple different string hashes to map the inputs into different integers, the LshBF schemes [22–25] apply locality sensitive hashing (LSH) [26] functions to directly transform high-dimensional vectors into serial real numbers by performing the dot product with the input dimensions and mapping similar vectors in the Euclidean space to near location(s). The LSH avoids “dimensional disasters” but results in a high FPP when querying memberships. To reduce the FPP, the LshBF-BF [23] adds a verification BF to further disperse vectors. According to the central limited theorem [36], the LSH shrinks all elements of the set around the mean. For example, when the LSH satisfies the standard normal distribution, approximately 68.5% of the elements gather between the negative and positive variance after mapping, which makes it more suitable for approximate nearest neighbours search.

## 3. Methods and structure

### 3.1 Standard Bloom filter and Counter Bloom filter

**Definition 1.** Bloom filter (BF) **[1]**. A Bloom filter contains *k* independent string hash functions *h*_*j*_(*j* = 1,…,*k*) and an array of *m* bits initiated to 0. By projecting *k* hashes, the BF stores *n* elements of a set *S* (*V*_1_,*V*_2_…*V*_*n*_) into the bit array. For *h*_*j*_ (*j* = 1,…,*k*) and *V*_*i*_(*i*≤*n*), the bit *h*_*j*_(*V*_*i*_)%*m* is set to 1. A bit can be set to 1 multiple times, but only the first change has an effect. Given a query *q*, if *h*_*j*_(*q*)%*m* = 1 for all *h*_*j*_ (*j* = 1,…,*k*), the *q* is accepted as a member of *S* with a false positive probability (FPP).

**Fig 1:**
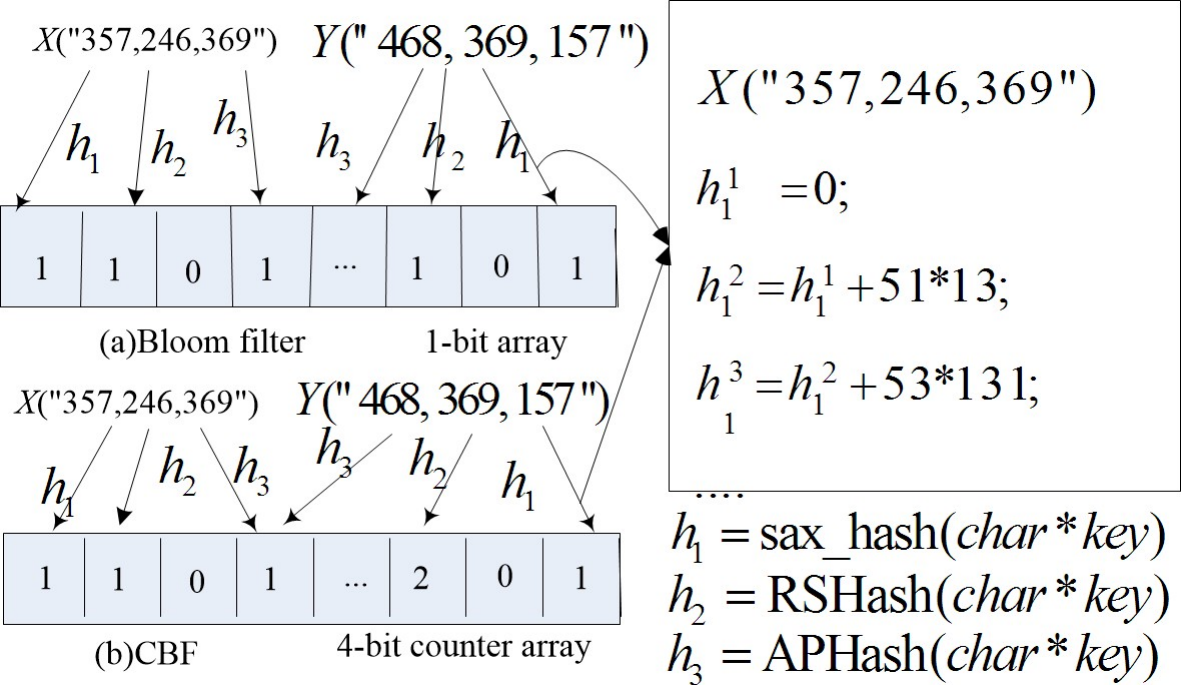
Bloom filter and counting Bloom filter.

The BF assumes that each *h*_*j*_ (*j* = 1,…,*k*) can randomly and uniformly map elements. Usually, *h*_*j*_ is a string hash [21], such as sax_hash and RSHash. By repeatedly iterating every character of *V*_*i*_, *h*_*j*_ obtains an integer in the range of [0−(2^31^-1)] (32 bits length) as the random hash fingerprint of *V*_*i*_. For example, given two vectors *X*(357,246,369) and *Y*(468,369,157), the sax_hash function (*h*_1_) uses ASCII codes of characters '3','5','7',',','2'… of *X* to iteratively compute a random integer. Then, the counter *h*_1_(*X*)%*m* is added with 1, as shown in Fig 1.

### 3.2 Prime high dimensional Bloom filter

**Fig 2:**
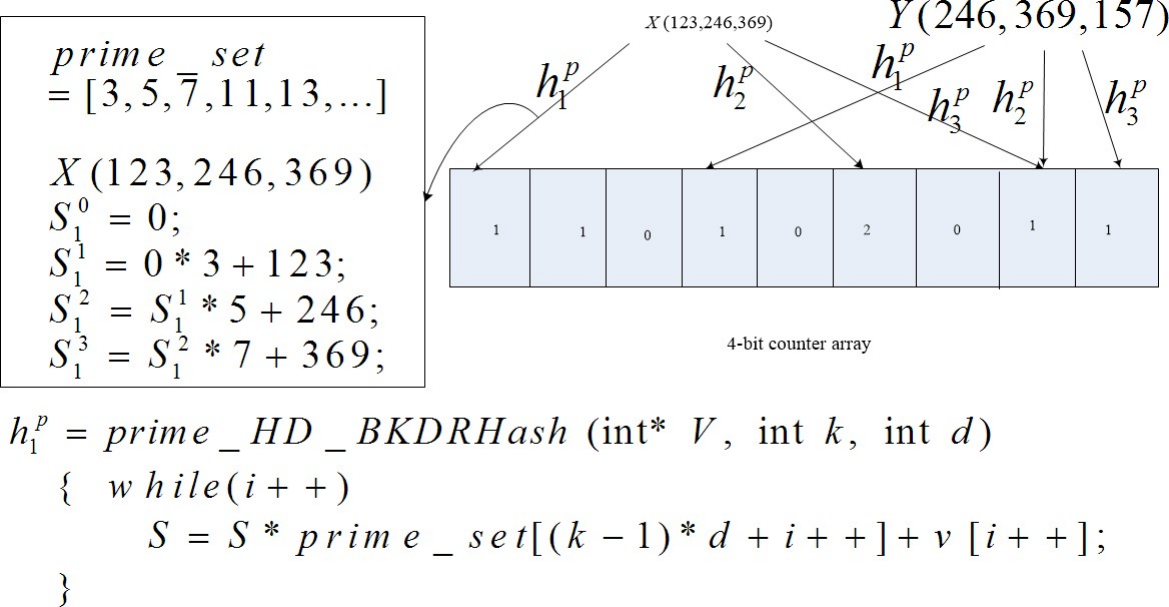
P-HDBF and prime_HDBKDRHash.

To address numerical vectors with high dimensions in their original formats other than strings, a new uniform hash function family, denoted as Prime_HD_BKDRHash, is proposed. Based on the unified Prime_HD_BKDRHash and a counter array, a new BF called P-HDBF is built, as shown in Fig 2.

**(1) Prime_HD_BKDRHash.** It originates from the BKDRHash function [27] and prime numbers. Given a prime number set *P* = [3,5,7,11,13,17…](except of 2) and a *d* dimensional numerical vector *V*(*v*_1_,…,*v*_*d*_), Prime_HD_BKDRHash considers *V* as a *d* dimensional vector. By iteratively computing *h*^*p*^ = *S*_*i*_ = ∏*p*_*i*-1_⋅*S*_*i*−1_+*v*_*i*_ (*i* = 1…*d*), *d* dimensions contribute to the last hash value. Although the *jth* operation and the (*j*+1)*th* operation are same, the corresponding prime numbers are different. Therefore, *h*_*j*_(*V*) and *h*_*j*+1_(*V*) will get different hash values (details in section 4.1).

**(2)A counter array (CA).** The array of P-HDBF contains *m* counters and each counter occupies 4 bits, which is enough to defend against the FNP brought by deleting elements [12]. When *k* random integers are calculated by *k* Prime_HD_BKDRHash functions, the counter *h*_*j*_(*V*)%*m*(1≤*j*≤*k*) of the CA is added to 1.

## 4. Theoretical analysis

The BF structure can work well only when the hashes can randomly and uniformly project all elements, since it is the basis of the BF. Therefore, this section will discuss the hash family- Prime_H-D_BKDRHash which is based on BKDRHash [27], and demonstrate why it is effective in the projection and query of high-dimensional vectors. The definition, proof and algorithm are shown as follows.

### 4.1 Prime_HD_ BKDRHash

**Definition 2.** A family *H*^*p*^ = {*h*^*p*^:*R*^*d*^→*U*} of functions is called a prime high-dimensional BKDRHash (Prime_HD_BKDRHash), if ∀*V*∈*R*^*d*^,*V*(*v*_1_,…,*v*_*d*_) and a prime number set *P*(*p*_1_,…,*p*_*l*_)(*l*∈∞) (without 2), such that *h*^*p*^ = *S*_*i*_ = ∏*p*_*i*-1_⋅*S*_*i*−1_+*v*_*i*_ (*i* = 1…*d*).

**Theorem 1.** By *h*^*p*^ mapping, all vectors *V*_*j*_(*v*_1_,…,*v*_*d*_) with *d* dimensions in a set will be randomly and uniformly projected to different integers.

**Proof.** Since *h*^*p*^ = *S*_*i*_ = ∏*p*_*i*-1_⋅*S*_*i*−1_+*v*_*i*_ (*i* = 1…*d*), then

$$\begin{array}{l} S_{d}=(p_{2}\cdot \ldots \cdot p_{i}\cdot \ldots \cdot p_{d})v_{1}+(p_{3}\cdot \ldots \cdot p_{i}\cdot \ldots \cdot p_{d})v_{2}\\ \;+\ldots +(p_{i+1}\cdot \ldots \cdot p_{d})v_{i}+\ldots +v_{d}\\ \;=v_{1}\prod_{i=2}^{d}p_{i}+v_{2}\prod_{i=3}^{d}p_{i}+\ldots +v_{i}\prod_{i=i+1}^{d}p_{i}+\ldots +v_{d} \end{array}.$$ (1)

Let *p*_*i*_<2^32^, 0<*v*_*i*_≪2^32^ (*i* = 1,…,*d*), *α*_*d*_ = 1 and *α*_*d*−1_ = *p*_*d*_⋅*α*_*d*_. For any *i* = 1,…,*d*−1, *α*_*i*_ = *p*_*i*+1_⋅*α*_*i*+1_>0 and *α*_*j*_>*α*_*i*_(*j*>i). If *α*_*i*_<2^32^ and any *α*_*j*_⋅*v*_*j*_>2^32^ (*j*>*i*), *α*_*j*_⋅*v*_*j*_ overflows, and the overflow part will be discarded by the 32-bit CPU. Since *p*_*j*_∈*P* (without 2), *p*_*i*_ is an odd number, and the multiplication of odd numbers is still an odd number. Since ∃*x*'∈*N*, $\prod_{j=j+1}^{d}p_{j}=2x^{'}+1$. Meanwhile, *v*_*j*_∈*N* and 0<*v*_*i*_≪2^32^, therefore

$$\begin{array}{l} a_{i}\cdot v_{j}=v_{j}\prod_{j=j+1}^{d}p_{j}\\ \;=v_{j}(2x^{'}+1)(x^{'}\in N)\\ \;=v_{j}\cdot 2x^{'}+v_{j}\\ \;=2^{32}+\beta_{j}v_{j} \end{array}.$$ (2)

If *α*_*i*_<2^32^ and *α*_*i*−1_ = *p*_*i*_⋅*α*_*i*_>2^32^, there always exists *z*_*i*−1_ = 2*x*+1(*x*∈*N*), which makes

$$\begin{array}{l} \alpha_{i-1}\cdot v_{i-1}=(2^{32}+z_{i-1})\cdot v_{i-1}\\ \;=2^{32}\cdot v_{i-1}+z_{i-1}\cdot v_{i-1} \end{array},$$ (3)

and

$$\begin{array}{l} \alpha_{i-2}\cdot v_{i-2}=p_{i-1}\cdot \alpha_{i-1}\cdot v_{i-2}\\ \;=p_{i-1}\cdot (2^{32}+z_{i-1})\cdot v_{i-2}\\ \;=p_{i-1}\cdot 2^{32}\cdot v_{i-2}+p_{i-1}\cdot z_{i-1}\cdot v_{i-2} \end{array}.$$ (4)

The same operations are applied on other fields from *α*_1_⋅*v*_1_ to *α*_*i*−3_⋅*v*_*i*−3_. For *v*_*i*_∈*N*, all 2^32^⋅*v*_*i*−1_ and *p*_*j*_⋅…⋅*p*_*i*−1_⋅2^32^⋅*v*_*j*_(1≤*j*<*i*−2) will be discarded by the 32-bit CPU due to overflow. Then,

$$\begin{array}{l} S_{d}=(p_{1}\cdot \ldots \cdot p_{i-1}\cdot z_{i-1})v_{1}+(p_{2}\cdot \ldots \cdot p_{i-1}\cdot z_{i-1})v_{2}\\ \;+\ldots p_{i-1}z_{i-1}\cdot v_{i-2}+z_{i-1}\cdot v_{i-1}+\alpha_{i}v_{i}+\ldots \beta_{j}v_{j}+\ldots +v_{d} \end{array}.$$ (5)

If *p*_*j*_…⋅*p*_*i*−1_⋅z_*i*-1_ = 2^32^+*z*(*j*<*i*), the CPU will iteratively discard the overflows. After multiple iterations,

$$\begin{array}{l} S_{d}=z_{1}\cdot v_{1}+\ldots +\ldots p_{i-2}z_{i-1}\cdot v_{i-2}\\ \;+z_{i-1}\cdot v_{i-1}+\alpha_{i}v_{i}+\ldots \beta_{j}v_{j}+\ldots +v_{d}^{'} \end{array},$$ (6)

where, for example, *p*_*i*_∈*P*, *z*_*i*−1_ = 2*x*+1(*x*∈*N*), *z*_1_ = *p*_1_⋅*p*_2_…⋅*p*_*l*_⋅*z*_*i*−1_ *z*_2_ = *p*_2_…⋅*p*_*l*_⋅*z*_i−1_. Therefore, *p*_1_*p*_2_…*z*_*i*−1_≠*p*_2_…*z*_*i*−1_≠*p*_*i*−2_*z*_*i*−1_≠*z*_*i*−1_≠*α*_1_ ≠ *β*_*j*_…<2^32^. Next, according to congruence theory [37],

$$\begin{array}{l} S_{d}\%m=(z_{1}\cdot v_{1}+\ldots +p_{i-2}\cdot z_{i-1}\cdot v_{i-2}+z_{i-1}\cdot v_{i-1}+\alpha_{i}v_{i}+\ldots \beta_{j}v_{j}+\ldots +v_{d})\%m\\ \;=(z_{1}\cdot v_{1}\%m\ldots p_{i-2}\cdot z_{i-1}\cdot v_{i-2}\%m+z_{i-1}\cdot v_{i-1}\%m+\alpha_{i}v_{i}\%m+\ldots \beta_{j}v_{j}\%m+\ldots +v_{d}\%m)\%m \end{array}.$$ (7)

**Worst case:** Since *p*_*i*_∈*P* (without 2), *p*_*i*_ is an odd number, and the multiplication of odd numbers is still an odd number. Let *n*_*i*_ be positive integers. Then, $\prod_{i=1}p_{i}=2n_{1}+1$, $\prod_{i=2}p_{i}=2n_{2}+1$ and $\prod_{i=i}p_{i}=2n_{i}+1$. Therefore,

$$S_{d}=(2n_{1}+1)v_{1}+(2n_{2}+1)v_{2}+\ldots +(2n_{i}+1)v_{i}+\ldots +v_{d}.$$ (8)

For *S*_*d*_, if 2*n*_*i*_≥2^32^, the CPU will discard the overflow part. At worst, for all *i*, 2*n*_*i*_ = *k*_*i*_2^32^(*k*_*i*_∈*N*), then *S*_*d*_ = *v*_1_+*v*_2_+…+*v*_*i*_+…+*v*_*d*_.

The function *S*_*d*_%*m* maps the *S*_*d*_ into the counter array, according to congruence theory [37].

$$\begin{array}{l} S_{d}\%m=\left(v_{1}+\ldots +v_{i}+\ldots +v_{d}\right)\%m\\ \;=\left(v_{1}\%m+\ldots +v_{i}\%m+\ldots +v_{d}\%m\right)\%m \end{array}.$$ (9)

From formulas (7) and (9), even in the worst case, every dimension *v*_*i*_ contributes to *S*_*d*_%*m*. In fact, from formula (7), all of the coefficients are odd numbers and they are different. For a well-selected *m*, different *v*_*i*_ will have different contributions to the final result, and the change of a *v*_*i*_ will change *S*_*d*_%*m*. Therefore, *h*^*p*^ satisfies the avalanche effect of hash functions [38] and can be regard as a uniform hash function.

**Lemma 1.** For a vector *V*, functions $h_{i}^{p}$ and $h_{j}^{p}$ are independently selected from the Prime_HD_BKDRhash family. There exist $h_{i}^{p}(V)\neq h_{j}^{p}(V)$ and $h_{i}^{p}(V)\%m\neq h_{j}^{p}(V)\%m$.

**Explain.** For any two hash functions $h_{i}^{p}$ and $h_{j}^{p}$, $h_{i}^{p}(V)\neq h_{j}^{p}(V)$. For simplicity, let *d* = 3, *V*(*v*_1_,*v*_2_,*v*_3_), $h_{1}^{p}$ and $h_{2}^{P}$. From formula (1),

$$\begin{array}{c} S_{{}_{3}}^{h_{1}^{p}}=(p_{1}p_{2}p_{3})v_{1}+(p_{2}p_{3})v_{2}+p_{3}v_{3}\\ S_{{}_{3}}^{h_{2}^{p}}=(p_{4}p_{5}p_{6})v_{1}+(p_{5}p_{6})v_{2}+p_{6}v_{3}) \end{array},$$ (10)

where *p*_1_<*p*_2_<*p*_3_<*p*_4_<*p*_5_<*p*_6_ and *p*_*i*_∈*P*. Therefore, $S_{{}_{3}}^{h_{1}^{p}}\neq S_{{}_{3}}^{h_{2}^{p}}$ and $h_{1}^{p}(V)\neq h_{2}^{p}(V)$.

Let *q*_1_ and *q*_2_ be quotients and *r*_1_ and *r*_2_ be remainders. $S_{{}_{3}}^{h_{1}^{p}}$ and $S_{{}_{3}}^{h_{2}^{p}}$ mod *m* are

$$\begin{array}{c} r_{1}=S_{{}_{3}}^{h_{1}^{p}}\%m=((p_{1}p_{2}p_{3})v_{1}+(p_{2}p_{3})v_{2}+p_{3}v_{3})\%m\\ r_{2}=S_{{}_{3}}^{h_{2}^{p}}\%m=((p_{4}p_{5}p_{6})v_{1}+(p_{5}p_{6})v_{2}+p_{6}v_{3})\%m \end{array}.$$ (11)

For a proper *m* and *r*_1_≠*r*_2_, $h_{1}^{p}$ and $h_{2}^{p}$ can scatter vectors into different positions. Without the loss of generality, 3 dimensions expand to *d* dimensions and $h_{1}^{p}$ and $h_{2}^{p}$ spread to $h_{i}^{p}$ and $h_{j}^{p}$. For well selected prime numbers, the worst case of formula (9) can be avoided, since $h_{i}^{p}(V)\neq h_{j}^{p}(V)$ and *r*_*i*_≠*r*_*j*_,

$$\begin{array}{c} r_{i}=S_{{}_{d}}^{h_{i}^{p}}\%m=((p_{i*d+1}p_{i*d+2}\ldots p_{i*d+d})v_{1}+(p_{i*d+t}\ldots p_{d})v_{t}+p_{d}v_{d})\%m\\ r_{j}=S_{{}_{d}}^{h_{j}^{p}}\%m=((p_{j*d+1}p_{j*d+2}\ldots p_{j*d+d})v_{1}+(p_{j*d+t}\ldots p_{d})v_{t}+p_{d}v_{d})\%m \end{array}.$$ (12)

### 4.2 Algorithm

From the above discussion, the Prime_HD_BKDRhash functions can randomly and uniformly map high-dimensional vectors to integers and Algorithms 1 combined with Fig 2 demonstrate the working process.

**Algorithm 1**.

unsigned int Prime_HD_BKDRHash (int* V, int k, int d)

{

1. unsigned int prime_set = [3,5,7,11,13,17 …];

2. unsigned int S = 0, i = 0;

3. while (*V)

4.         S = prime_set [k*d+i++] *S+ (*V++);

5. return S&0x0FFFFFFF;

}

The input parameters contain the vector *V*, dimensions *d* and the *kth* hash function. After *d* loops, lines 3 and 4 of Algorithm 1 obtain $h_{k}^{p}=S_{{}_{i}}=\prod p_{i-1}\cdot S_{i-1}+v_{i}\;(i=1\ldots d)$. By performing a bitwise AND on *S*_*i*_ and 0x0FFFFFFF, the *kth* Prime_HD_BKDRHash transforms the vector *V* into an integer that ranges from [0−(2^32^−1)]. Since different hash functions adopt different prime numbers, the return integers are different.

## 5. Performances

In section 4, we have demonstrated that the Prime_HD_ BKDRHash can randomly and uniformly scatter the high-dimensional vectors of a set to integers in the range of 0 to 2^32^−1. Therefore, the P-HDBF satisfies the theory of the BF, including all parameters and their relationships.

### 5.1 False positive probability (FPP), m, n, k and false negative probability (FNP)

**FPP.** Let there be *k* hash functions, a counter array of size *m* and a set containing *n* vectors with *d* numerical dimensions. After the *n* vectors are mapped onto the P-HDBF, the false positive probability of the P-HDBF is [15].

$$f_{P-HDBF}=(1-{\left(1‑1/m\right)}^{nk}{)}^{k}\approx (1-e^{-\frac{nk}{m}}{)}^{k}.$$ (13)

**Counters.** For fixed *k*, *n* and FPP, **t**he counters that the P-HDBF requires are

$$m=-\frac{kn}{\ln(1-f_{P-HDBF}{}^{1/k})}.$$ (14)

**Maximum cardinality.** For fixed *m*,*k* and FPP, the maximum number of the vectors the P-HDBF can represent is

$$n_{0}=-\frac{\ln(1-e^{\ln\frac{f_{0}}{k}})\cdot m}{k}.$$ (15)

**Minimum number of hash functions.** For fixed *m*, *n* and FPP, the minimum number of hash functions is

$$k_{\min}=\ln\;2(\frac{m}{n}).$$ (16)

**False negative probability (FNP).** The FNP of the P-HDBF is

$$fnp_{P-HDBF}=0.$$ (17)

### 5.2 Time complexity

For a hash *h*_*j*_ and a query *q*, every numerical dimension *q*_*i*_ will participate in the computation. By computing the *k* hashes, the P-HDBF obtains *k* integers. Next, by mapping *h*_*j*_(*q*)%*m*, the P-HDBF checks whether the corresponding *k* counters are greater than or equal to 0. If any counter is 0, we know that the query is not in the set. If all counters are larger than 0, the query is determined as a member of the set with a small FPP. For a set of *n* elements with *d* dimensions, its initialization time complexity is

O(kn). (18)

The time complexity of insertion/deletion/query of a vector is

O(k). (19)

## 6. Experiment

### 6.1. Dataset and setting

To verify the effectiveness of the P-HDBF on high-dimensional numeric vectors, this paper adopts 3 picture datasets, including Colour [39], Sift [40] and Gist [40], used in most experiments. On these datasets, we compare the performances of the P-HDBF with the CBF, PBF-HT and PBF-BF. The CBF is the classical method in all variants and the PBF-HT and PBF-BF support the query of multiple dimensions. The Colour includes 70,000(70K) vectors with 32 dimensions, and the values are expanded to positive integers. The Sift and Gist contain 100,000 (100K) vectors with 128 and 960 dimensions, respectively, and values of dimensions are all positive integers. All query vectors are different from the samples and are set to 10,000(10K). The experiments were conducted on a computer with an Intel Xeon E5-2603 v3 and 16GB RAM.

### 6.2 Distribution and entropy

The key of the BF is that the data can be randomly and uniformly projected by hash functions. To verify this performance, we first introduce information entropy of the array in the BF after Prime_HD_BKDRHash mapping. Information entropy can describe the randomness of a system, and a larger entropy indicates a greater dispersed state. Let *v*' be the number of the elements allocated in a counter of the array, and *n* and *m* be the size of the set and the array, respectively. Then, the proportion of the vectors allocated in the counter can be calculated by *p*≈*v*'/*kn*, and the entropy of all counters is defined as follows.

$$E=\sum_{m}-p\log\;p\approx \sum_{m}-(v'/kn)\log(v'/kn).$$ (20)

**Fig 3:**
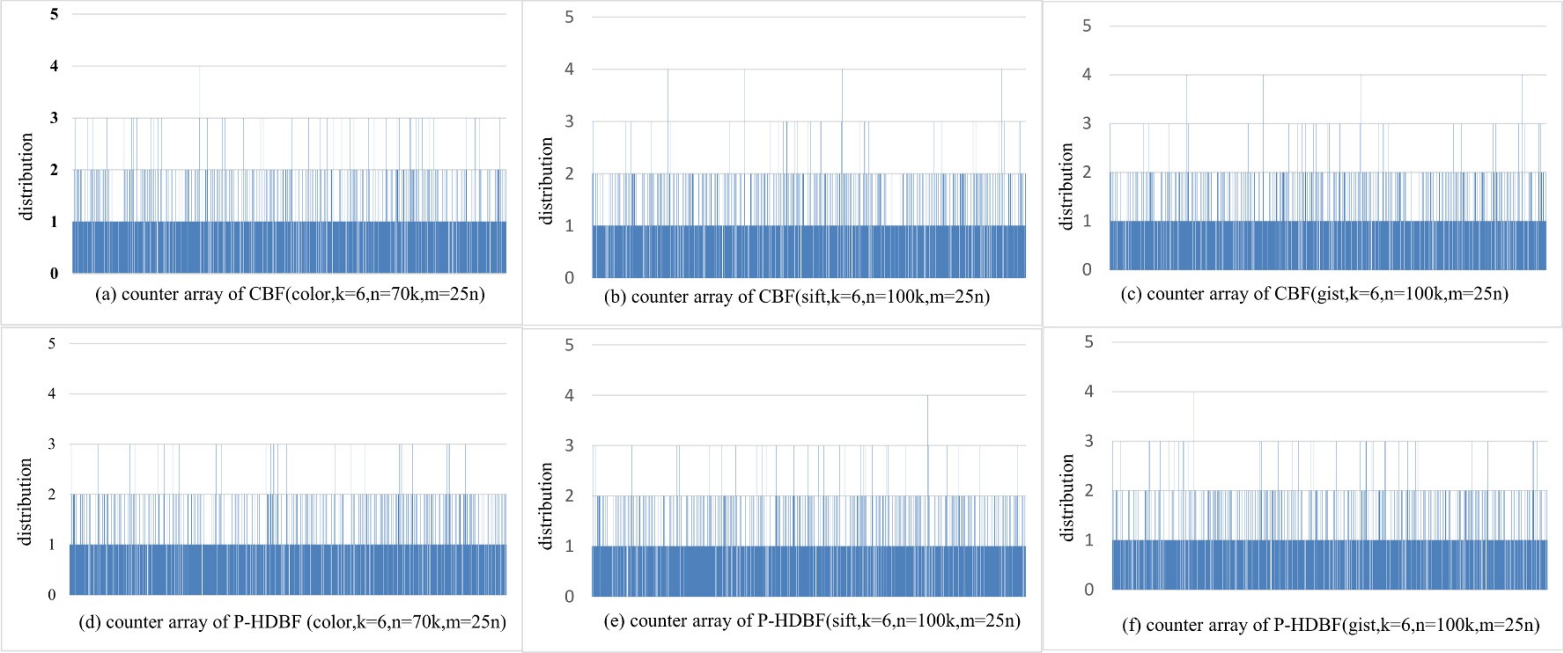
Number of vectors allocated in different counters of the CBF and the P-HDBF on colour (d = 32), Sift (d = 128) and Gist (d = 960).

**Fig 4:**
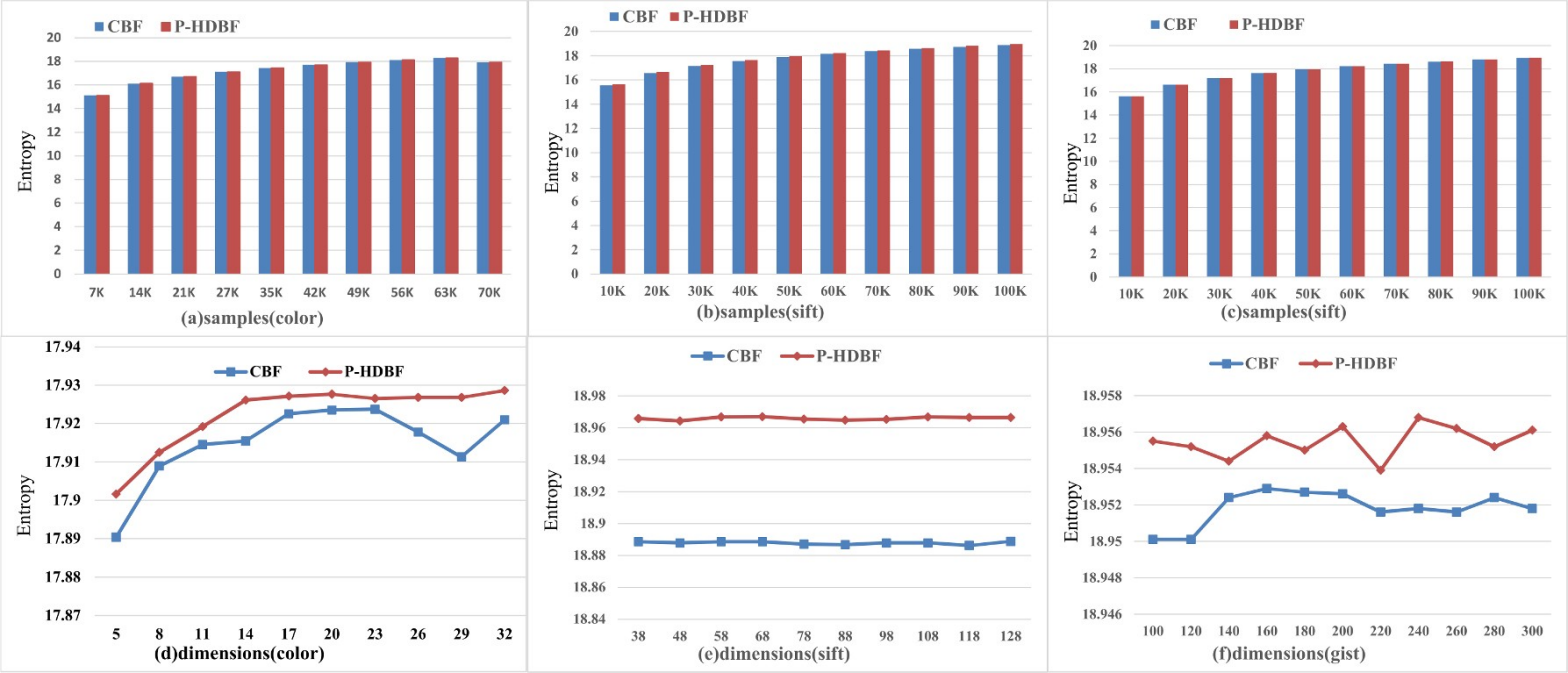
Entropy of the CBF and P-HDBF under different samples and dimensions.

Let *k* = 6 and *m* = 25*n*. Figs 3 and 4 display the number of the vectors allocated in different counters (denoted as distribution) and entropies of the P-HDBF and the CBF on the 3 datasets. As Fig 3 shows, the distribution of the P-HDBF is similar to the CBF, which implies that the vectors are uniformly allocated in different counters. Fig 4 shows that the entropy of the P-HDBF is slightly larger than that of the CBF under different samples and dimensions, especially in a high-dimensional space. From the view of the entropy, larger entropy means better discretization and less collision. Figs 3 and 4 reflect that the Prime_HD_BKDRHash can scatter vectors more randomly and uniformly, especially for the high-dimensional vectors of a big data set (on Gist with d = 960). This implies that the FPP of the query after mapping by the uniform Prime_HD_BKDRHash will be less than that of multiple string hashes. The uniform Prime_HD_BKDRHash can project the numerical vectors as the inputs of the original formats and substitute multiple different string hashes of the BF.

### 6.3 Relationships of the FPP, n, m, d and k

**Fig 5:**
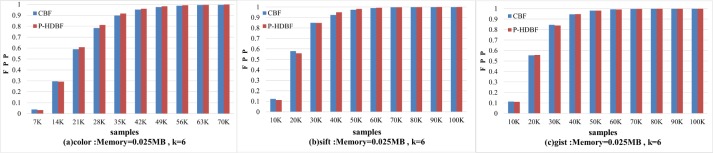
FPPs of the CBF and P-HDBF with an increasing n.

**Fig 6:**
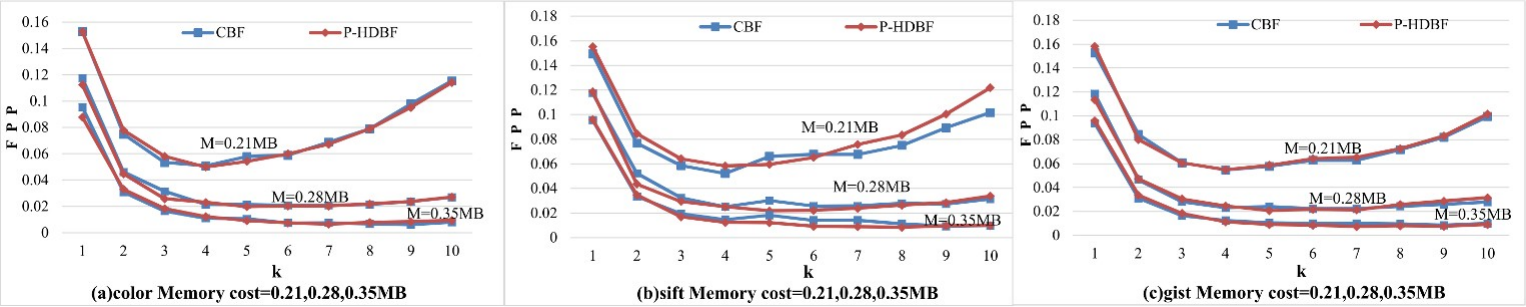
FPPs of the CBF and the P-HDBF under different k and memory costs.

**Fig 7:**
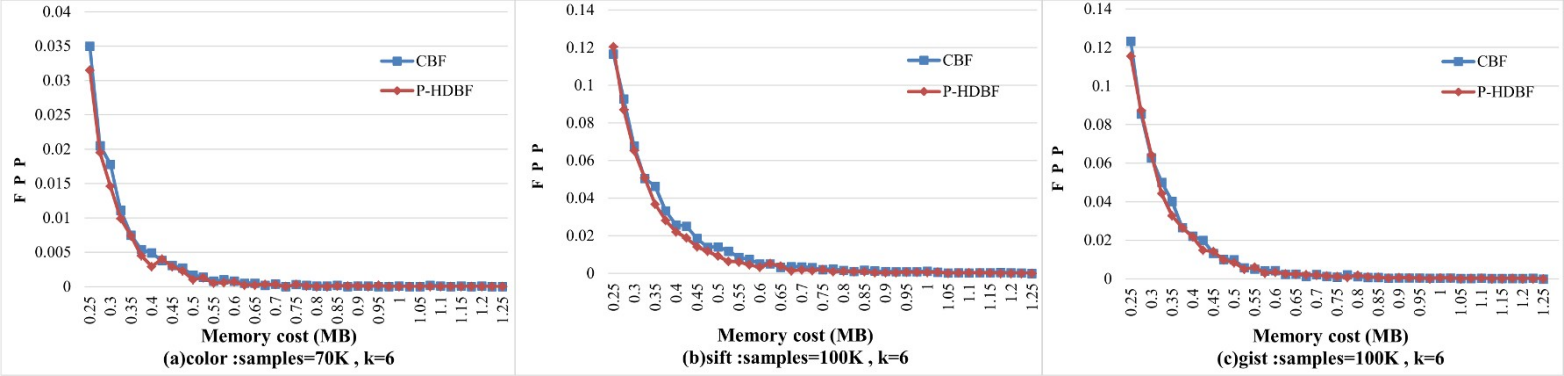
FPPs of the CBF and P-HDBF with the memory increase.

This section will show whether the P-HDBF is consistent with the theory of the Bloom filter, and the indicators include the FPP, n, m, k and their relationships. The CBF, as a classical BF, is applied for comparison with the P-HDBF, and the P-HDBF should show the same tendencies as the CBF. By fixing one or two parameter(s) in turn, Figs 5, 6 and 7 show the FPPs’ changes with other parameters changing. For the fixed memory costs (m) and the number of the hash functions (k), the increased collision rate causes the FPP growing. Let *m* = 50*k* and *k* = 6. Fig 5 displays the increased tendencies of the FPPs as the cardinality of the set increases, even to 100%.

Then, by fixing memory costs (0.21 MB, 0.28 MB, and 0.35 MB), Fig 6 demonstrates the FPPs as *k* increases on the 3 datasets. For the fixed number of samples (n) and memory costs (m), the number of the hash functions (k) determines the FPP. Firstly, the FPP will decrease as *k* grows and reach to a minimum value, then the increasing collisions will result in a low FPP. With *k* rising, both FPPs sharply decrease, reach a minimum value, and then increase slowly, which is consistent with the theory of the BF.

Lastly, for *k* = 6, Fig 7 displays the similar changes in the FPPs of the CBF and the P-HDBF as *m* increases from 5*n* to 25*n*. For fixed *k* and *n*, the FPP will be decided by memories allocated to them, and a large *m* can effectively reduce the FPPs.

**Fig 8:**
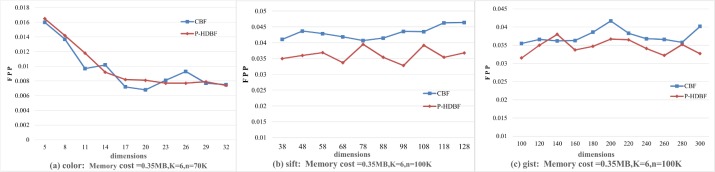
FPPs of the CBF and P-HDBF under different dimensions.

To further observe the performance of the P-HDBF in a high dimensional space, an extra experiment is added. Let *n* = 70*K*(100*K*,100*K*), *k* = 6 and the memory be 0.35 MB. Fig 8 demonstrates the changes of the FPPs with increasing dimensions. The FPPs of the P-HDBF are lower than those of the CBF, especially in certain high dimensional cases.

For different *m*, *n* and *k*, the FPPs’ changes of the CBF and the P-HDBF are almost the same. Even the performance of the P-HDBF is better than the CBF, which implies that the P-HDBF can replace the CBF to process high-dimensional vectors. Meanwhile, the FPPs’ changes of the P-HDBF are consistent with the theory in section 5. Next, we will continue to compare the performance of the P-HDBF with other methods.

### 6.4 Compared with other methods

**Fig 9:**
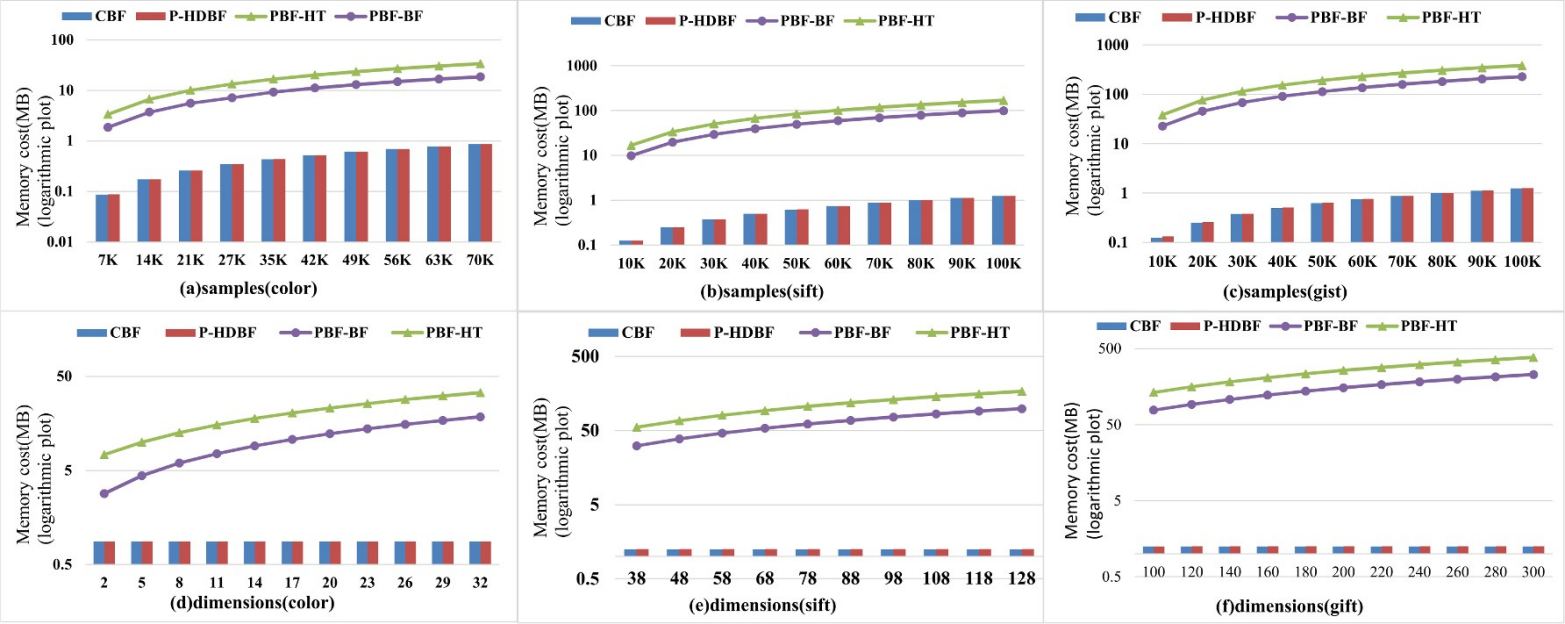
Memory costs under different samples and dimensions.

Let *FPP*⊂[0.0001−0.0005], *m* = 25*n* and *k* = 6. This paper compares the memory usages of the CBF, PBF-BF and PBF-HT with the P-HDBF on 3 datasets, as shown in Fig 9. For the fixed FPP, the CBF and the P-HDBF have memory overheads. However, the memory costs of the PBF-BF and the PBF-HT grow with increased sample sizes and dimensions.

**Fig 10:**
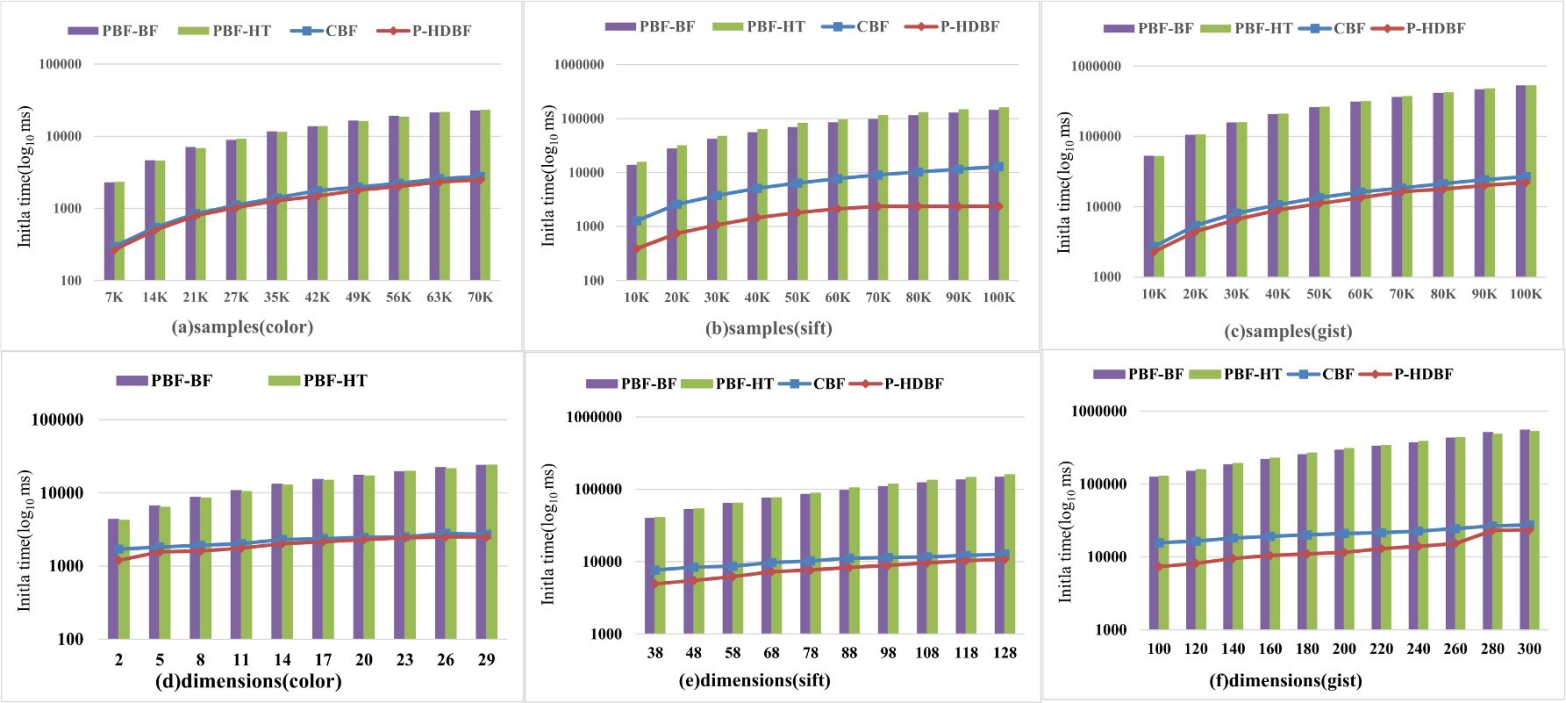
Average initiation time of the PBF-HT, PBF-BF, CBF and P-HDBF with *FPP*⊂[0.0001−0.0005] and *k* = 6.

**Fig 11:**
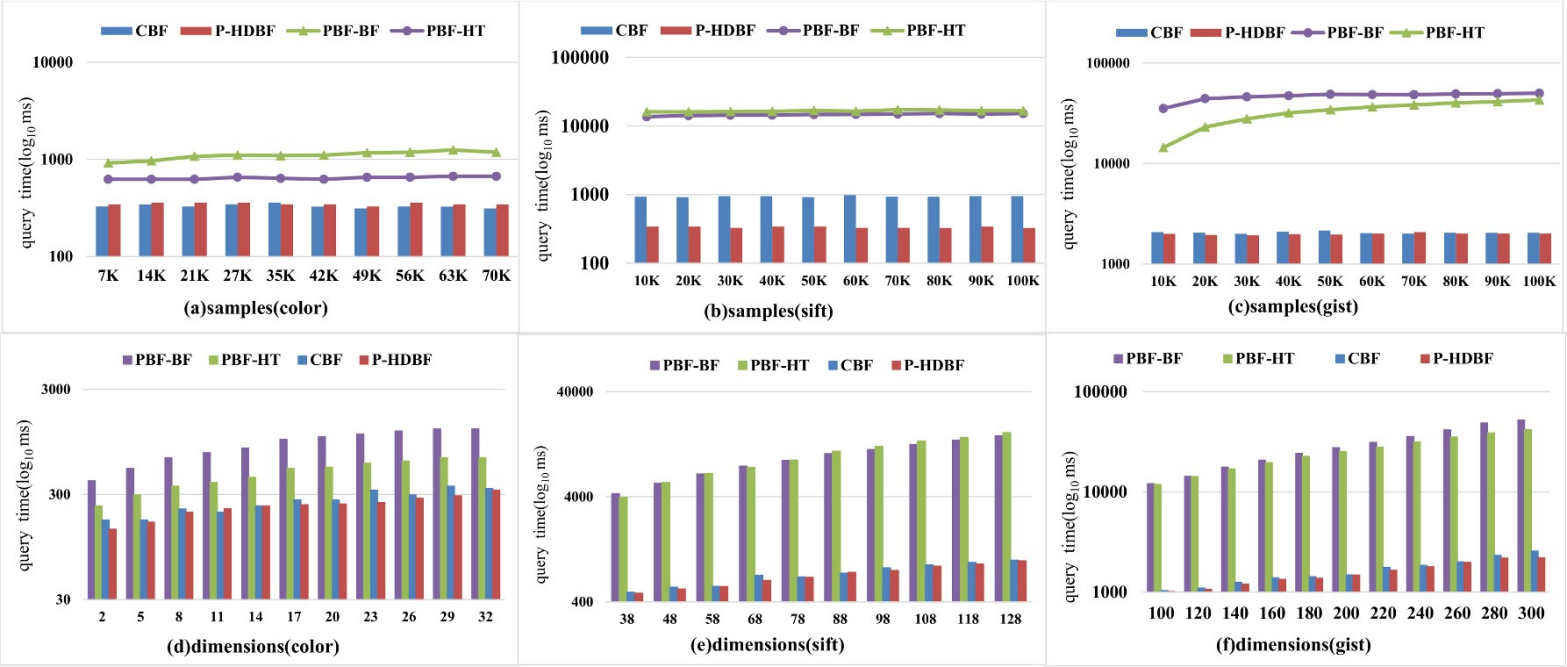
Average query delays of the PBF-HT, PBF-BF,CBF and P-HDBF with *FPP*⊂[0.0001−0.0005] and *k* = 6.

Figs 10 and 11 exhibit the average initiation and query time of different schemes under 10K query vectors. Since these schemes need to split all vectors and project them into the storing arrays, the initiation and query times will continue to increase with larger samples and more dimensions. Compared with the PBF-BF and PBF-HT, the CBF and the P-HDBF only require dividing the dimensions and computing the hash values. Therefore, their initiation and query times increase slowly with more dimensions. The initiation time and query delays of the CBF and P-HDBF are far smaller than those of the PBF-BF and PBF-HT.

Therefore, for a given FPP and a dataset with high-dimensional vectors, the P-HDBF will be a better choice than the PBF-based schemes by avoiding a long member query delay and huge memory costs.

## 7. Conclusions

Regardless of the formats of the inputs, the traditional Bloom filters adopt multiple string hashes to implement memberships queries of a big data set. To map the inputs with numerical high dimensions in their original type(s), this paper proposes a uniform Prime_HD_BKDRHash function and establishes a P-HDBF structure, a new Bloom filter, to store and query members of a big data set with numerical dimensions. The unified Prime_HD_BKDRHash can randomly and uniformly project the inputs (other than multiple string hashes) into different integers. The performances and parameters of the P-HDBF have been theoretically discussed. The experiments show that the P-HDBF, as a substitute for the counting Bloom filter in high-dimensional numerical spaces, can obtain excellent data discretization and a good performance. Compared with the methods based on the parallel Bloom filters, the P-HDBF will not increase memory use or query delays as dimensions increase and can be used in applications with limited CPU and memory resources. The P_HDBF can be applied in some applications, such as identify the nuances of pictures.

## Abbreviations

*S* = (*V*_1_,…,*V*_*n*_): A set of *n* vectors for member query
*V*(*v*_1_…*v*_*d*_), *q*(*q*_1_…*q*_*d*_): A vector in *S* and a query *q* with *d* dimensions
*P*(*p*_1_…*p*_*l*_): A prime number set *P* and a prime number *p*_*i*_
H^p^: A Prime_HD_ BKDRHash function family with a function of *h_k_^P^* = *S_i_* = ∏*p*_*i*−1_⋅*S*_*i*−1_+*v_i_*(*i* = 1…*d*)
*f*_*P−HDBF*_, *fnp*_*P−HDBF*_: The false positive (negative) probability of P-HDBF.
m: The array size of P-HDBF.
k: The number of Prime_HD_ BKDRHash functions
